# Fabrication of Biomass Derived Pt-Ni Bimetallic Catalyst and Its Selective Hydrogenation for 4-Nitrostyrene

**DOI:** 10.3390/nano12172968

**Published:** 2022-08-27

**Authors:** Siyu Long, Lingyu Zhang, Zhuoyue Liu, Huibin Jiao, Aiwen Lei, Wei Gong, Xianglin Pei

**Affiliations:** 1 School of Materials and Architectural Engineering, Guizhou Normal University, Guiyang 550025, China; 2 Guizhou Key Laboratory of Inorganic Nonmetallic Functional Materials, Guizhou Normal University, Guiyang 550025, China; 3 School of Materials Science and Engineering, Guizhou Minzu University, Guiyang 550025, China; 4 College of Chemistry and Molecular Sciences, Wuhan University, Wuhan 430072, China

**Keywords:** biomass chitin, Pt-Ni nanoparticles, supported catalyst, hydrogenation

## Abstract

The hydrogenation products of aromatic molecules with reducible groups (such as C=C, NO_2_, C=O, etc.) are relatively critical intermediate compounds in fine chemicals, but how to accurately reduce only specific groups is still challenging. In this work, a bimetallic Pt-Ni/Chitin catalyst was prepared for the first time by using renewable biomass resource chitin as support. As the carrier, the chitin was constructed into porous nanofibrous microspheres through the sol-gel strategy, which was favorable for the adhesion of nano-metals and the exchange of reactive substances due to its large surface area, porous structure, and rich functional groups. Then the Pt-Ni/Chitin catalyst was applied to selective hydrogenation with the model substrate of 4-nitrostyrene. As the highly dispersed Pt-Ni NPs with abundant exposed active sites and the synergistic effect of bimetals, the Pt-Ni/Chitin catalyst could efficiently and selectively hydrogenate only NO_2_ or C=C with yields of ~99% and TOF of 660 h^−1^, as well as good stability. This utilization of biomass resources to build catalyst materials would be important for the green and sustainable chemistry.

## 1. Introduction

Catalytic hydrogenation based on transition metal catalysts is of great significance in both laboratory and industry and has been widely developed [1,2]. Typically, the hydrogenation products of arylamines (from nitroaromatics) and aralkyls (from aryl olefins) are all the critical intermediate compounds in fine chemicals, and have a wide range of applications in pharmaceutical, chemical, petroleum, cosmetic, and other fields [3,4]. However, the ability of aromatic molecules with reducible functional groups (such as C=C, NO_2_, C=O, etc.) to be activated or reduced is different [5,6]. How to accurately reduce the appointed groups is still a challenge. For example, in most reported literatures, Pd or Pt based supported catalysts showed excellent catalytic activity in hydrogenation of aromatic molecules with reducible C=C and NO_2_ groups [7,8,9]. However, due to the strong reduction ability of Pd or Pt nano-metals, the specific ability to reduce only NO_2_ (or C=C) is weak, i.e., the chemoselectivity is poor [10,11]. To improve the chemoselectivity, various attempts have been conducted, such as changing the hydrogen source, selecting different nano-metal catalysts, constructing alloy catalysts, designing the structure of catalyst supports, etc. [1,2,3,4,10].

Generally, the catalyst support materials play important roles in catalysis, which can disperse the nano-metal particles to improve their utilization, and contribute to the recycling of catalysts [12,13]. In particular, some support materials can interact with nano-metals, either to firmly anchor the nano-metals or change the coordination environment or surface electronic structure of nano-metals, thereby significantly improving the performance of the nano-metal catalysts [14,15]. In recent years, biomass resource chitin has gradually attracted widespread attention as a catalyst carrier material [16]. As the second largest natural polymer on Earth after cellulose, chitin has a wide range of sources and low price, which is suitable for industrial mass production [17,18,19]. Not only that, the chitin molecular chains are rich in characteristic functional groups such as hydroxyl and acetamide, which can coordinate with nano-metals to anchor them firmly, as well as adjusting the coordination environment of the nano-metal catalysts to improve the catalytic performance [18]. Importantly, based on the rigidity of the chitin molecular chain, chitin in nature mostly presents a multi-level micro-nano structure, which can efficiently disperse nano-metals and improve the utilization rate of metal catalysts [19,20]. In addition, the rigid structure of chitin leads to its good thermal and chemical stability, which is also an advantage as a catalyst support [16,17].

In our previous study, we have used the sol-gel strategy to construct a chitin microsphere woven by nanofibrous, which further gave the chitin a larger specific surface area [21]. Herein, going one step further, we used the above microspheres as support to fabricate a series of Pt-based monometallic/bimetallic catalysts and used them for the chemoselective hydrogenation of 4-nitrostyrene. Owing to the rich functional groups and large surface area of chitin microspheres, the nano-metals were well tightly dispersed on the chitin microspheres, proved by various characterizations. Notably, due to the synergistic effect of bimetals, the chitin supported Pt-Ni catalyst could efficiently and selectively hydrogenate only NO_2_ or C=C on aromatic molecules with good yields by changing the reaction conditions. By using the biomass resource chitin as a carrier, which not only broadens the application chitin materials, but also enables such biomass-based catalysts to be used in photocatalysis, electrocatalysis, and other fields.

## 2. Experimental Section

### 2.1. Materials

Chitin was purchased from Zhejiang Golden Shell Biochemical Co., Ltd. (Taizhou, China). Metal salts such as chloroplatinic acid hexahydrate (AR, Pt ≥ 37.5%, H_2_PtCl_6_·6H_2_O), Nickel(II) acetate tetrahydrate (99.9%, Ni(CH_3_COO)_2_·4H_2_O), Palladium acetate (99.9%, Pd(OAc)_2_), and 4-nitrostyrene (>98%, stabilized with TBC) were purchased from Aladdin (Aladdin Inc., Shanghai, China). Commercial Pt/C (20%) and nano-Pt (99.9%) were purchased from Macklin (Macklin Inc., Shanghai, China). Span 85 (Aladdin Inc., Shanghai, China), Tween 85 (Aladdin Inc., Shanghai, China), and isooctane (99.7%, Tianjin Damao Chemical Reagent Factory, Tianjing, China) were used as received. All other reagents, such as isopropanol, toluene, and hydrochloric acid, were obtained from various commercial sources and used without further purification.

### 2.2. Synthesis of the Chitin Supported Nano-Metal Catalysts

#### 2.2.1. Synthesis of the Chitin Microspheres

Chitin powders purchased from Zhejiang Golden Shell Biochemistry Co., Ltd. (Zhejiang, China) were dispersed in 100 g NaOH/urea water system (11 wt%/4 wt%) and placed in a low-temperature cold trap for freezing/thawing 3–4 times to obtain a clear, transparent, and mobile chitin solution. The above chitin solution was added into a mixed solution of isooctane, span 85, and tween 85, then fully stirred under ice conditions for 2 h through the suspension emulsion strategy. Then, the reaction mixture temperature was heated to 90 °C, and maintained for 5 min. Subsequently, pH of the solution was adjusted to neutral with 10% HCl to obtain the chitin emulsion microspheres. The chitin emulsion microspheres were further washed with ethanol and water, filtered, and dried for later use.

#### 2.2.2. Synthesis of the Chitin Supported Monometallic Catalysts

Taking Pt/Chitin as an example, a certain amount of H_2_PtCl_6_·6H_2_O ([Pt]:chitin = 1.8 wt%) dissolved in deionized water was added dropwise to 200 mg of chitin microspheres. The mixed solution was stirred for 4 h at room temperature, then washed with deionized water for several times, subsequently reduced with NaBH_4_ and separated by filtration to obtain the monometallic Pt/Chitin catalyst. The other monometallic catalysts such as Fe/Chitin, Co/Chitin, Ni/Chitin, Cu/Chitin, etc. in this work were prepared using the above method.

#### 2.2.3. Synthesis of the Chitin Supported Bimetallic Catalysts

Taking Pt-Ni/Chitin (3:1) as an example, a certain proportion of well-mixed H_2_PtCl_6_·6H_2_O and Ni(CH_3_COO)_2_ ·4H_2_O ([Pt]:[Ni] = 3:1, mol%) aqueous solution was stirred for 0.5 h at room temperature, and then added dropwise to 200 mg of chitin microspheres ([Pt-Ni]:chitin = 1.8 wt%). The mixed solution was stirred for 4 h at room temperature, then washed with deionized water for several times, subsequently reduced with NaBH_4_, and separated by filtration to obtain the bimetallic Pt-Ni/Chitin catalyst. The other bimetallic catalysts such as Pt-Fe/Chitin, Pt-Co/Chitin, Pt-Ni/Chitin, Pt-Cu/Chitin, etc. in this work were prepared using the above method.

#### 2.2.4. Selective Hydrogenation for 4-Nitrostyrene

Selective hydrogenation of C=C to C-C for 4-nitrostyrene: The hydrogenation of 4-nitrostyrene was used to evaluate the catalytic activity of the chitin supported catalysts. In a typical experiment, 0.5 mmol of 4-nitrostyrene, 4 mg of nano-metal catalysts, and 5 mL of solvents were added to a 25 mL reaction flask, the reactor was charged with H_2_ with pressure of 1 bar, and the reaction was stirred in a water bath for several hours. The reaction mixture was analyzed with gas chromatography (GC) to analyze the reaction mixture with biphenyl as the internal standard substance. Control experiments were performed with Pd/Chitin, Fe/Chitin, Co/Chitin, Ni/Chitin, Cu/Chitin, etc.

Selective hydrogenation of NO_2_ to NH_2_ for 4-nitrostyrene: In a typical procedure, 0.5 mmol of 4-nitrostyrene, 2 mmol of NaBH_4_, 4 mg of Pt-Ni/Chitin, and 5 mL of solvents were added to a 25 mL reaction flask, and the mixture was magnetically stirred with magnetic stirring for several hours. After the reaction was complete, the reaction mixture was analyzed with GC to analyze the reaction mixture with biphenyl as the internal standard substance.

### 2.3. Characterization

X-ray diffraction (XRD) pattern was recorded by an X-ray powder diffraction (XRD, Rigaku Miniflex600 with Cu Kα radiation, λ = 1.5406 Å, Tokyo, Japan). Infrared spectroscopy was carried out by using a Fourier transform infrared spectrometer (FT-IR, PerkinElmer Corporation/model 1600, MA, USA). Nitrogen adsorption and desorption measurements were recorded by a Micromeritics AsAp2020 (Norcross, GA, USA). X-ray photoelectron spectroscopy (XPS) was collected on a VG Multi Lab 2000 system with a monochromatic A1 *Kα* X-ray source (Thermo Fisher scientific ESCALAB 250Xi, MA, USA). GC yields were recorded through a Varian GC 2014 gas chromatography instrument with an FID detector. Scanning electron microscopy (SEM) images were observed by field emission scanning electron microscopy (FESEM, Zeiss SUPRA 55 Sapphire, Oberkochen, Bartenburg State, Germany) at an accelerating voltage of 5 kV. Transmission electron microscopy (TEM) images were collected on a JEM-2010 (HT) electron microscope (JEOL, Tokyo, Japan) with an accelerating voltage of 200 kV. Before the TEM observation, the sample was thoroughly ground, then we impregnated it with ethanol and dropped the suspension onto a copper grid. 

## 3. Results and Discussion

### 3.1. Formation of the Chitin-Supported Metal Catalysts and Its Selective Hydrogenation of C=C to C-C

**Figure 1:**
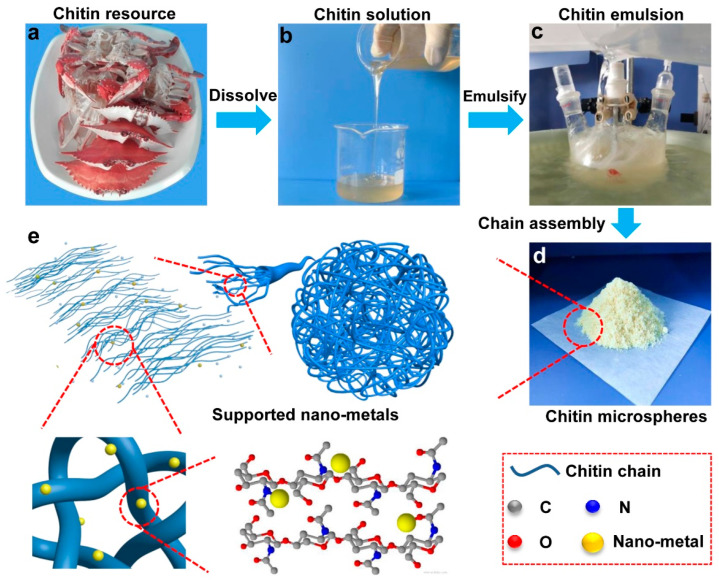
Schematic diagram of the formation process for the chitin supported nano-metal catalyst: The chitin resource (**a**). The chitin solution (**b**). The chitin emulsion (**c**). The chitin microspheres (**d**). The chitin microspheres supported nano-metals (**e**).

According to the sol-gel strategy, chitin raw materials acquired from the market were physically dispersed in the aqueous NaOH/urea system at low temperature to break up its large numbers of inter- and intra-molecular hydrogen bonds. Then, we used isooctane as the oil phase, Span 85 and Tween 85 as the surfactant, and obtained a nanofibrous chitin microsphere via emulsion method by sol-gel process. During the sol-gel process, the hydrogen bonds between the chitin molecular chains recombine, resulting in the aggregation and re-arrangement of chitin molecular chains, and then form spherical nanofibrous microspheres through the suspension emulsion method [21]. Further, the chitin microspheres were used as supports and impregnated with metal precursors, which were subsequently reduced with reductant to obtain the chitin supported nano-metal catalysts. The schematic diagram of the formation process is shown in Figure 1.

Before figuring out the structure of the chitin supported nano-metal catalysts, we directly used these catalysts for catalytic chemical reactions. Usually, it is difficult for aromatic molecules with reducible C=C or NO_2_ groups to selectively hydrogenate only C=C or NO_2_ groups [3,4,6]; for example, the aralkyl products, which play important roles in large chemical production, petroleum, cosmetic, organic intermediates, material chemistry, medicinal chemistry, etc. Herein, first of all, the aim of selectively hydrogenating only C=C was attempted, and the 4-nitrostyrene was used as the model substrate to evaluate the catalytic activity of these different chitin supported catalysts. First, we took the most common hydrogenation catalysts of Pd and Pt species reported in the literature as examples [22]. To select the best reaction conditions, the reaction solvent (Table S1 entries 1–8) and temperature (Table S1 entries 8–11) were screened. As shown in Table 1, by using isopropyl alcohol as the solvent, the Pt/Chitin catalyst could selectively hydrogenate only C=C with 4-nitroethylbenzene yield of 87% at room temperature in 24 h or even longer (Table 1, entry 1; Table S1, entry 8). Although the selectivity was good, the reaction time was too long. In contrast, the Pd/Chitin catalyst could give 92% yield of 4-nitroethylbenzene and 3% yield of 4-aminoethylbenzene in 1 h (Table 1, entry 2). However, the 4-nitroethylbenzene gradually turned into 4-aminoethylbenzene with an increase in the reaction time (Table 1, entry 3). Combining the high activity of Pd metal and the high selectivity of Pt metal, Pt-Pd/Chitin bimetallic catalyst was designed, which was effective in improving the selectivity of 4-nitroethylbenzene (Table 1, entry 5), but also did not solve the problem of -NO_2_ to NH_2_ group by extending the reaction time (Table 1, entry 6). To further improve the selectivity of 4-nitroethylbenzene and taking into account the consideration of low cost, a series of bimetallic catalysts combining Pt with common non-precious metals (Fe, Co, Ni, Cu) were designed [15,23,24,25,26]. It could be seen that the Pt-Ni/Chitin bimetallic catalyst could achieve the best selectivity of 4-nitroethylbenzene with a yield of 99% in 3 h or even longer time (Table 1, entries 7–10). As comparisons, the monometallic Fe/Chitin, Co/Chitin, Ni/Chitin, Cu/Chitin catalysts and blank chitin could not complete the conversion with poor activity (Table S2; Table 1, entry 4). In addition, the commercial Pt/C and nano-Pt catalysts could only give the target product yield of 42% and 5%, respectively (Table 1, entries 14–15), further suggesting the good catalytic activity of Pt-Ni/Chitin catalyst. Moreover, the optimal ratio of Pt to Ni in chitin was also explored, and it was found that the best selectivity of 4-nitroethylbenzene with yield of 99% was achieved when Pt:Ni was 3:1 (mol%, Table 1, entries 10–12), and the calculated TOF and TON were 660 h^−1^ and 1980, respectively (entry 10). After 48 h, the yield could also be maintained at 99%, effectively realizing the selective hydrogenation only C=C group (Table 1, entry 13). The probable mechanism for the selective hydrogenation of C=C to C-C was shown in Figure S5, the Pt-Ni NPs on the catalyst promoted the dissociation of hydrogen to form metal-H, which further attacked C=C to lead to the formation of saturated C-C. We believed that the Pt-Ni bimetallic catalyst formed by the introduction of Ni metal effectively promoted the selective hydrogenation of 4-nitrostyrene to 4-nitroethylbenzene.

### 3.2. Structure of the Pt-Ni catalyst

**Figure 2:**
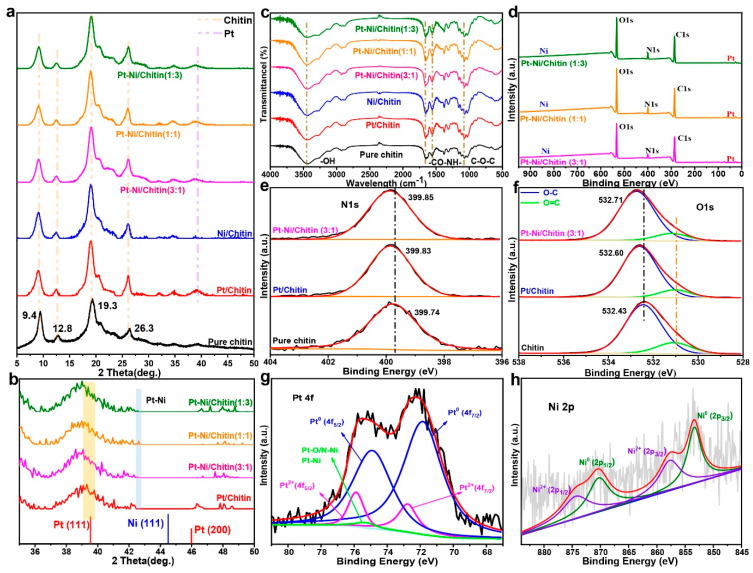
XRD patterns of the chitin supported catalysts (**a**). A partial enlargement of XRD patterns (**b**). FT-IR spectra of the chitin supported catalysts (**c**). The full scale XPS spectra of the Pt-Ni bimetallic catalysts (**d**). XPS spectra of N 1s (**e**), O 1s (**f**) of the pure chitin, Pt/Chitin, and Pt-Ni/Chitin. Pt 4f spectrum of the Pt-Ni/Chitin (**g**). Ni 2p spectrum of the Pt-Ni/Chitin (**h**).

The good catalytic activity and selectivity of Pt-Ni catalyst encourage us to explore its structure. In the study of the crystal structures, X-ray diffraction (XRD) patterns in Figure 2a showed that these chitin-supported catalysts all had distinct characteristic diffraction peaks at 2*θ* = 9.4°, 12.8°, 19.2°, and 26.3° after loading the nano-metals, and these peaks were attributed to the characteristic peaks of carrier chitin [17,27]. The new appeared characteristic peak at about 2*θ* = 39.5° was attributed to Pt (111) (JCPDS no. 04-0802) [28]. However, the peak attributed to Ni species could not be detected by XRD, which may be due to the small size or uniform dispersion of Ni particles [29]. Further, in the amplification analysis of 2*θ* = 35–50° ranges, a new weak peak of ~ 42.57° appeared (Figure 2b), which may arise from the formation of Pt-Ni alloy [27,28]. Bahrami et al. also reported a Pt-Ni/rGO counter electrode, and found that the reflection from (111) in the crystal planes was corresponding to Pt-Ni, with approximately 2*θ* = 42.64° [28]. In the Fourier transform infrared (FT-IR) spectra, as shown in Figure 2c, the blank chitin possessed characteristic absorption peaks of 3430 cm^−1^ attributed to -OH, 1670 or 1580 cm^−1^ attributed to amide, and 1070 cm^−1^ attributed to -C-O-C, etc. [17]. After the introduction of nano-metals, these chitin-supported catalysts likewise all contained above characteristic peaks with little change, further indicating the good stability of chitin [19].

To further demonstrate the chemical state of the Pt-Ni/Chitin catalyst, X-ray photoelectron spectroscopy (XPS) was conducted, and it could be seen that these Pt-Ni/Chitin catalysts contained C, O, N, Pt, and Ni elements (Figure 2d), further suggesting the successful introduction of Pt and Ni species. Moreover, in the Pt 4f of Pt-Ni/Chitin catalyst (Herein, taking catalyst of Pt-Ni/Chitin, Pt:Ni = 3:1 [mol%] as an example), the Pt 4f spectra in Figure 2g could mainly split into Pt 4f_7/2_ and Pt 4f_5/2_. The two peaks with binding energy (BE) of 71.89 and 74.81 eV were attributed to Pt^0^, and the BE of 72.77 and 75.88 eV were attributed to Pt^2+^. It was also noteworthy that there was a weak peak at 75.32 eV presumably attributed to Pt-N/O-Ni or Pt-Ni [25,26,27,28,29,30], which further proved the formation of Pt-Ni alloy. Pires et al. also reported a Pt-Ni bimetallic catalyst, and found that the Pt hydroxide and Pt oxide peaks were attributed to Pt-O-C or Pt-O-Ni bonds in the Pt 4f spectra, which suggested the Pt-C and Pt-Ni bonds and the formation of Pt-Ni alloy [30]. Similarly, in the Ni 2p spectra, the characteristic peaks in Pt-Ni/Chitin corresponding to Ni^0^ (853.37 eV, 870.27 eV) and Ni^2+^ (857.53 eV, 874.20 eV) were also be found (Figure 2h) [11,27]. The interaction between chitin and nano-metals was also investigated by the binding energy of XPS spectrum; here the catalyst of Pt-Ni/Chitin (Pt:Ni = 3:1 [mol%]) was still used as an example, and the spectrum had been calibrated with a C 1s of 284.80 eV. As shown in Figure 2e, the peak of N 1s spectra in blank chitin shifted from 399.74 to 399.85 eV after the loading of Pt-Ni NPs. In addition, in the O 1s spectra, the two peaks at 532.43 (O-C) and 530.94 eV (O=C) also shifted to higher values after the addition of Pt-Ni NPs (Figure 2f). In contrast, the C 1s spectra of the Pt-Ni/Chitin had almost no shift relative to that of blank chitin (Figure S1). Overall, the binding energy of N 1s and O 1s in Pt-Ni/Chitin moved toward a higher binding energy compared to that of blank chitin, which indicated that there was an interaction between N/O and nano-metals [19,25,27].

**Figure 3:**
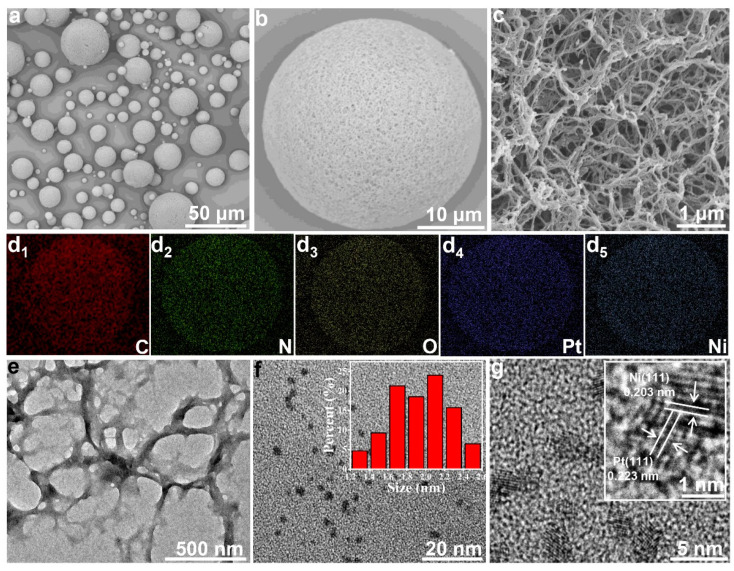
SEM images of the chitin microspheres (**a**–**c**). EDX mapping of the Pt-Ni/Chitin (**d_1_**−**d_5_**). TEM images of the Pt-Ni/Chitin (**e**–**g**), inset (**f**) with the particle size distribution, inset (**g**) with the HR-TEM image of a single Pt-Ni particle.

Further, nitrogen adsorption and desorption measurement of the chitin microspheres indicated that the isotherms were type IV with an H3 hysteresis loop (Figure S2), and the Brunauer-Emmett-Teller (BET) surface area of the chitin microspheres was calculated as 253 m^2^g^−1^. Meanwhile, the Barret-Joyner-Halenda (BJH) theory pore-size distribution data (inset in Figure S2) revealed that the pores presented in chitin microspheres were mainly mesoporous [17]. Scanning electron microscopy (SEM) images in Figure 3a–c revealed that the chitin microspheres were woven by nanofibrous with nanoporous structure [31]. We believed that the large specific surface area and rich pore structure were favorable for the adhesion of nano-metals and the exchange of reactive substances. After loading the nano-metals, the nanofibrous morphology of chitin microspheres was well maintained (Figure S3) [4], indicating that the nano-metal loading did not affect the structure of the chitin microspheres. Energy dispersive X-ray spectroscopy (EDX) mapping analysis of Pt-Ni/Chitin in Figure 3(d_1_–d_5_) showed that the Pt-Ni NPs were uniformly dispersed throughout the chitin microspheres relative to the framework C/O/N elements [27,29,32]. 

To determine the morphology of the Pt-Ni NPs immobilized on chitin, transmission electron microscopy (TEM) was conducted, which further the proved the porous structure of chitin microspheres and revealed that the Pt-Ni NPs (catalyst of Pt-Ni/Chitin, Pt:Ni = 3:1 [mol%]) were uniformly distributed on the surface of the support without obvious aggregation (Figure 3e–g), and the average particle size of Pt-Ni NPs was about 2.1 nm (inset in Figure 3e). Moreover, high resolution TEM (HR-TEM) images in Figure 3g and Figure S4 showed that in a typical dark spot (i.e., nano-metal particle), clear lattices attributed to the Pt (111) (d = 0.223 nm) and Ni (111) (d = 0.203 nm) phases were observed [26,33,34], further suggesting the formation of Pt-Ni NPs, which was consistent with the results of XRD and XPS. Duan et al. reported am Ag-Fe bimetallic catalyst and used it for degradation of 4-nitrophenol. In a typical dark spot of nano-metal particle, they found the characteristic lattice spacing of Ag (111) (d = 0.235 nm), Ag (200) (d = 0.203 nm) and Fe (311) (d = 0.251 nm), Fe (511) (d = 0.162 nm), respectively, indicating the formation of Ag-Fe alloy [33]. Similarly, Zhang, et al. synthesized a PtNix/CNTs catalyst, and proved the Pt-Ni alloy by characteristic lattice spacing of Pt (111) (d = 0.223 nm) and NiO (d = 0.241 nm) in a typical dark spot of nano-metal particle [29]. Evidently, a highly dispersed Pt-Ni bimetallic catalyst supported on chitin was successfully fabricated.

### 3.3. Selective Hydrogenation of NO_2_ to NH_2_ for the Pt-Ni/Chitin Catalyst

It is also very important to convert nitroalkenes into aminoalkene compounds, and the aminoalkene compounds are of great significance in the synthesis of pharmaceutical intermediates, pesticides, natural products, etc. [6,8]. Further, we also attempted the Pt-Ni/Chitin catalyst for selective hydrogenation of NO_2_ to NH_2_ with the model substrate of 4-nitrostyrene. It was striking that the Pt-Ni/Chitin catalyst could selectively hydrogenate only NO_2_ to NH_2_, i.e., 4-nitrostyrene to 4-aminostyrene, when the hydrogen source was changed to NaBH_4_ (Table 2). In screening the solvents, toluene:H_2_O = 1:1 was determined to be the optimal solvent for the reaction, which could reach 99% yield of 4-aminostyrene (Table 2, entries 1–9), and the calculated TOF and TON were 660 h^−1^ and 1980, respectively (entry 9). When screening the effect of temperature on the reaction, it was found that the product yield increased with increasing the temperature and peaked at 60 °C, and the lower yield of the target product at high temperature may be due to the occurrence of side reactions caused by high temperature (Table 2, entries 9–13). Commercial Pt/C, commercial nano-Pt and un-supported H_2_PtCl_6_▪6H_2_O catalysts were also used as controls, as shown in Table S3. The commercial Pt/C and nano-Pt catalysts showed lower target product yields compared to the Pt-Ni/Chitin catalyst. Although the in situ reduced Pt in this system by using un-supported H_2_PtCl_6_▪6H_2_O could give target product yield of 57% (Table S3, entry 3), it could not be reused for lack of carrier. Furthermore, the optimal ratio of Pt to Ni in chitin for the selective hydrogenation only NO_2_ to NH_2_ were also evaluated. As shown in Table S4 (entries 1–3), the Pt-Ni/Chitin with Pt:Ni ratio of 3:1 (mol%) could also give the best yield. Notably, the Pt-Ni/Chitin catalyst was active for the selective hydrogenation of NO_2_ or C=C group by adjusting the reaction condition. The proposed mechanism for selective hydrogenation of NO_2_ to NH_2_ is shown in Figure S6, as the interaction between the center of the Pt-Ni/Chitin catalyst and H. The activated hydrogen consequently interacted with the nitro compounds to generate the hydroxylamine intermediate. Following the removal of water, the aniline product and the Pt-Ni/Chitin would be released to complete the catalytic cycle. 

**Figure 4:**
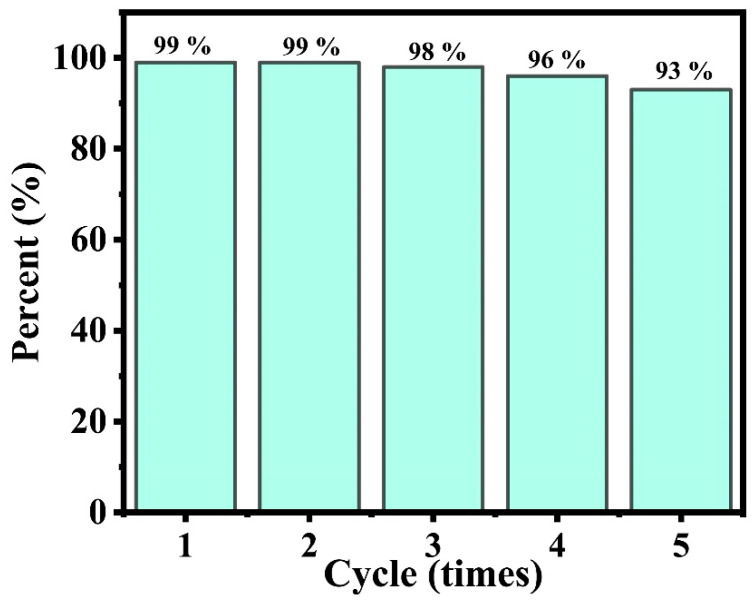
The reusability of the Pt-Ni/Chitin catalyst for selective hydrogenation of 4-nitrostyrene to 4-aminostyrene.

For supported catalysts, the cycle stability was also an important indicator. Here, we performed multiple reaction cycles on the Pt-Ni/Chitin catalyst with the model reaction of 4-nitrostyrene to 4-aminostyrene, and it could be seen that the catalyst showed good stability in five consecutive cycle reactions (Figure 4). The yield of the target product could still maintain 93% after 5 runs, indicating that the catalyst we obtained had excellent reusability. TEM image in Figure S7 for the five reused Pt-Ni/Chitin catalysts showed that the Pt-Ni NPs were slightly agglomerated, but there was no obvious aggregation. Inductive coupled plasma emission spectrometer (ICP) results revealed that the Pt to Ni loading in Pt-Ni/Chitin changed little from the initial 1.21, 0.39 wt% to 1.12, 0.36 wt% after five runs, respectively (Table S5), suggesting that very little nano-metal was lost to the reaction solution. Evidently, the Pt-Ni/Chitin bimetallic catalyst had good activity, selectivity, and stability on the hydrogenation of NO_2_ or C=C. The good catalytic performance of the Pt-Ni/Chitin bimetallic catalyst may be due to the following reasons: (1) The porous chitin microspheres woven by nanofibrous could provide abundant adhesion sites for nano-metals, as well the exchange of reactive substances. (2) The rich functional groups of -OH, NH_2_, etc. in chitin microspheres could interact with nano-metals to anchor them tightly, thus enhancing the stability of the catalyst. (3) The tiny and highly dispersed nano-metal particles could expose more active sites to improve the reactivity. (4) The formed bimetallic catalyst with synergistic effect of bimetals facilitated the selective hydrogenation of the catalyst.

## 4. Conclusions

In summary, for the first time, a highly dispersed Pt-Ni bimetallic catalyst anchored on porous nanofibrous chitin microspheres was successfully constructed, confirmed by diverse physicochemical characterizations. As the carrier, the chitin microspheres possessed abundant functional groups of hydroxyl and acetamide, as well large surface area, which promoted the high dispersion of Pt-Ni NPs. Importantly, the formed Pt-Ni bimetallic catalyst with synergistic effect of bimetals could significantly improve the performance of the catalyst. The Pt-Ni/Chitin catalyst was used for selective hydrogenation of 4-nitrostyrene, which showed good catalytic activity, selectivity, and cycling stability by adjusting the reaction conditions. This research enriches the application of chitin-based materials by using residual biomass resource chitin as the catalyst carrier, as well as promotes the development of green and sustainable chemistry. 

## Figures and Tables

**Table 1:** Selective hydrogenation of C=C to C-C for 4-nitrostyrene *^a^*.

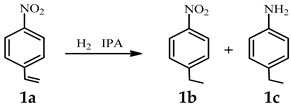				
Entry	Catalyst	Time (h)	Yield *^b^* (%)	Yield *^c^* (%)
1	Pt/Chitin	24	87	Trace
2	Pd/Chitin	1	92	3
3	Pd/Chitin	4	46	53
4	Chitin	36	-	-
5	Pt-Pd/Chitin	2	99	-
6	Pt-Pd/Chitin	10	46	22
7	Pt-Fe/Chitin	3	87	-
8	Pt-Co/Chitin	10	82	-
9	Pt-Cu/Chitin	10	72	-
10	Pt-Ni/Chitin (3:1)	3	99	-
11	Pt-Ni/Chitin (1:1)	3	39	-
12	Pt-Ni/Chitin (1:3)	3	59	-
13	Pt-Ni/Chitin (3:1)	48	99	-
14	Commercial Pt/C *^d^*	24	42	Trace
15	Commercial nano-Pt *^e^*	24	5	-

*^a^* Reaction conditions: 4-nitrostyrene (0.5 mmol), nano-metal catalyst (4 mg), isopropanol (IPA, 5 mL), H_2_ (1 bar) at room temperature. *^b,c^* b was the yield of 1b, c was the yield of 1c, and the yield was GC yield. *^d,e^* The [Pt] content in these catalyst was the same as Pt-Ni/Chitin (3:1).

**Table 2:** Effect of solvent and temperature on the hydrogenation of 4-nitrostyrene *^a^*.

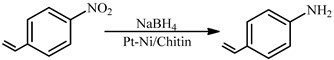				
Entry	Solvent	Temperature (°C)	Time (h)	Yield *^b^* (%)
1	Toluene	60	3	Trace
2	H_2_O	60	3	43
3	MeOH	60	3	24
4	THF	60	3	Trace
5	DCM	60	3	13
6	DMF	60	3	16
7	IPA	60	3	34
8	MeOH: H_2_O = 1:1	60	3	37
9	Toluene: H_2_O = 1:1	60	3	99
10	Toluene: H_2_O = 1:1	30	3	28
11	Toluene: H_2_O = 1:1	75	3	89
12	Toluene: H_2_O = 1:1	90	3	82
13	Toluene: H_2_O = 1:1	120	3	16

*^a^* Reaction conditions: 4-nitrostyrene (0.5 mmol), catalyst (4 mg), solvent (5 mL), NaBH_4_ (2 mmol). *^b^* The yield was GC yield.

## Data Availability

Data will be available upon request from the corresponding authors.

## Supplementary Materials

The following supporting information can be downloaded at: https://www.mdpi.com/article/10.3390/nano12172968/s1, Figure S1: XPS spectra of C 1s for pure chitin, Pt/Chitin and Pt-Ni/Chitin; Figure S2: Nitrogen adsorption & desorption isotherms and BJH pore-size distribution of the chitin microspheres; Figure S3: SEM image of the Pt-Ni/Chitin catalyst; Figure S4: HR-TEM images of an individual Pt-Ni alloy particle; Figure S5: The probable mechanism for the selective hydrogenation of C=C to C-C for 4-nitrostyrene; Figure S6: The probable mechanism for the selective hydrogenation of NO_2_ to NH2 for 4-nitrostyrene; Figure S7: TEM image of the Pt-Ni/Chitin after 5 runs, inset (f) with the particle size istribution; Table S1: Hydrogenation of 4-nitroethylene in various reaction conditions; Table S2: Some common non-precious metal catalysts; Table S3: Some commercial catalysts for hydrogenation of 4-nitrostyrene; Table S4: Effect of different ratios of Pt-Ni on the hydrogenation of 4-nitrostyrene; Table S5: The nano-metal content in Pt-Ni/Chitin (3:1) catalyst.

## References

[1] Pei Y., Moghadam B.R., Zhang X., Li Z., Wang J. (2020). Interface catalysis by Pt nanocluster@Ni_3_N for bifunctional hydrogen evolution and oxygen evolution. Mater. Chem. Front..

[2] Arenas L.F., Hadjigeorgiou G., Jones S., Van D.N., Hodgson D., Cruden A., de León C.P. (2020). Effect of airbrush type on sprayed platinum and platinum-cobalt catalyst inks: Benchmarking as PEMFC and performance in an electrochemical hydrogen pump. Int. J. Hydrogen Energy.

[3] Ding K., Hu Z., Zhang W.H., Liang J.X., Wang Y.M., Li H., Sun Z.J., Liang Q.L., Sun H.B. (2022). Bimetallic RhIn/ZIF-8 for the catalyic chemoselective hydrogenation of nitrostyrene: Exploration of natural selectivity of hydrogen sources and enhancing intrinsic selectivity. Micropor. Mesopor. Mat..

[4] Bai S.X., Bu L.Z., Shao Q., Zhu X., Huang X.Q. (2018). Multicomponent Pt-based zigzag nanowires as selectivity controllers for selective hydrogenation reactions. J. Am. Chem. Soc..

[5] Chen X., Shi C., Liang C. (2021). Highly selective catalysts for the hydrogenation of alkynols: A review. Chin. J. Catal..

[6] Shi Y., Wang Z., Liu C., Wu T.K., Liu R., Wu L. (2022). Surface synergetic effects of Pt clusters/monolayer Bi_2_MoO_6_ nanosheet for promoting the photocatalytic selective reduction of 4-nitrostyrene to 4-vinylaniline. Appl. Catal. B-Environ..

[7] Trandafir M.M., Pop L., Hădade N.D., Florea M., Garcia H. (2016). An adamantane-based COF: Stability, adsorption capability, and behaviour as a catalyst and support for Pd and Au for the hydrogenation of nitrostyrene. Catal. Sci. Technol..

[8] Zhou P., Li D.Z., Jin S.W., Chen S.H., Zhang Z.H. (2016). Catalytic transfer hydrogenation of nitro compounds into amines over magnetic graphene oxide supported Pd nanoparticles. Int. J. Hydrogen Energy.

[9] Martyna B., Krzysztof C., Wojciech K., Grzegorz B., Dariusz M., Beara M., Ryszard K., Beata Z. (2018). A comparison of hydrogen storage in Pt, Pd and Pt/Pd alloys loaded disordered mesoporous hollow carbon spheres. Nanomaterials.

[10] Sohn H., Xiao Q., Seubsai A., Ye Y., Lee J., Han H., Park S., Chen G., Lu Y. (2019). Thermally robust porous bimetallic (Ni_x_Pt_1–x_) alloy microcrystals within carbon framework: High-performance catalysts for oxygen reduction and hydrogenation reactions. ACS Appl. Mater. Interfaces.

[11] Gao H.G., Shi R., Shao Y.T., Liu Y., Zhu Y.F., Zhang J.G., Li L.Q. (2022). Catalysis derived from flower-like Ni MOF towards the hydrogen storage performance of magnesium hydride-science direct. Int. J. Hydrogen Energy.

[12] Theerthagiri J., Karuppasamy K., Lee S.J., Shwetharani R., Kim H.S., Pasha S.K.K., Ashokkumar M., Choi M.Y. (2022). Fundamentals and comprehensive insights on pulsed laser synthesis of advanced materials for diverse photo- and electrocatalytic applications. Light-Sci. Appl..

[13] Pei X.L., Deng Y., Li Y., Huang Y.G., Yuan K., Lee J.F., Chan T.S., Zhou J.P., Lei A.W., Zhang L.N. (2018). Size-controllable ultrafine palladium nanoparticles immobilized on calcined chitin microspheres as efficient and recyclable catalysts for hydrogenation. Nanoscale.

[14] Anaya-Castro F.D.J., Beltrán-Gastélum M., Soto O.M., Pérez-Sicairos S., Lin S.W., Trujillo-Navarrete B., Paraguay-Delgado F., Salazar-Gastélum L.J., Romero-Castañón T., Reynoso-Soto E. (2021). Ultra-Low Pt loading in PtCo catalysts for the hydrogen oxidation reaction: What role do Co nanoparticles play?. Nanomaterials.

[15] Li W., Chu X.S., Wang F., Dang Y.Y., Liu X.Y., Wang X.C., Wang C.Y. (2021). Enhanced cocatalyst-support interaction and promoted electron transfer of 3D porous g-C_3_N_4_/GO-M (Au, Pd, Pt) composite catalysts for hydrogen evolution. Appl. Catal. B-Environ..

[16] Jin T., Liu T., Lam E., Moores A. (2021). Chitin and chitosan on the nanoscale. Nanoscale Horizons.

[17] Pei X.L., Deng Y., Duan B., Chan T.S., Lee F.J., Lei A.W., Zhang L.N. (2018). Ultra-small Pd clusters supported by chitin nanowires as highly efficient catalysts. Nano Res..

[18] Zhang J.P., Lin F.Z., Yang L.J., He Z.Y., Huang X.S., Zhang D.W., Dong H. (2020). Ultrasmall Ru nanoparticles supported on chitin nanofibers for hydrogen production from NaBH_4_ hydrolysis. Chin. Chem. Lett..

[19] Pei X.L., Jiao H.B., Fu H., Yin X.G., Luo D., Long S.Y., Gong W., Zhang L.N. (2020). Facile construction of a highly dispersed Pt nanocatalyst anchored on biomass-derived N/O-doped carbon nanofibrous microspheres and its catalytic hydrogenation. ACS Appl. Mater. Inter..

[20] Hou W.X., Zhao Q.Z., Liu L. (2020). Selective conversion of chitin to levulinic acid catalyzed by ionic liquids: Distinctive effect of N-acetyl groups. Green Chem..

[21] Duan B., Zheng X., Xia Z., Fan X., Guo L., Liu J., Wang Y., Ye Q., Zhang L.N. (2015). Highly biocompatible nanofibrous microspheres self-assembled from chitin in NaOH/urea aqueous solution as cell carriers. Angew. Chem. Int. Ed..

[22] Nishida Y., Sato K., Chaudhari C., Yamada H., Toriyama T., Yamamoto T., Matsumura S., Aspera S.M., Nakanishi H., Haneda M. (2022). Nitrile hydrogenation to secondary amines under ambient conditions over palladium–platinum random alloy nanoparticles. Catal. Sci. Technol..

[23] Duan Y., Yu Z.Y., Yang L., Zheng L.R., Zhang C.T., Yang X.T., Gao F.Y., Zhang X.L., Yu X., Liu R. (2020). Bimetallic nickel-molybdenum/tungsten nanoalloys for high-efficiency hydrogen oxidation catalysis in alkaline electrolytes. Nat. Commun..

[24] Yu Y., Lee S.J., Theerthagiri J., Fonseca S., Pinto L.M.C., Maia G., Choi M.Y. (2022). Reconciling of experimental and theoretical insights on the electroactive behavior of C/Ni nanoparticles with AuPt alloys for hydrogen evolution efficiency and non-enzymatic sensor. Chem. Eng. J..

[25] Mcbride F., Hodgson A. (2018). The reactivity of water and OH on Pt-Ni(111) films. Phys. Chem. Chem. Phys..

[26] Yu Y., Lee S.J., Theerthagiri J., Lee Y., Choi M.Y. (2022). Architecting the AuPt alloys for hydrazine oxidation as an anolyte in fuel cell: Comparative analysis of hydrazine splitting and water splitting for energy-saving H_2_ generation. Appl. Catal. B-Environ..

[27] Guo L.H., Tian Y.J., He X.Y., Qiao C.Z., Liu G.Z. (2022). Hydrodeoxygenation of phenolics over uniformly dispersed Pt-Ni alloys supported by self-pillared ZSM-5 nanosheets. Fuel.

[28] Bahrami A., Kazeminezhad I., Abdi Y. (2019). Pt-Ni/rGO counter electrode: Electrocatalytic activity for dye-sensitized solar cell. Superlattices Microst..

[29] Zhang J.K., Zheng X.H., Yu W., Feng X., Qin Y. (2022). Unravelling the synergy in platinum-nickel bimetal catalysts designed by atomic layer deposition for efficient hydrolytic dehydrogenation of ammonia borane. Appl. Catal. B-Environ..

[30] Pires M.D.S., Haye E., Zubiaur A., Job N., Pireaux J.J., Houssiau L., Busby Y. (2019). Defective Pt-Ni/graphene nanomaterials by simultaneous or sequential treatments of organometallic precursors by low-pressure oxygen plasma. Plasma Processes Polym..

[31] Zhou J., An W., Wang Z. (2019). Hydrodeoxygenation of phenol over Ni-based bimetallic single-atom surface alloys: Mechanism, kinetics and descriptor. Catal. Sci. Technol..

[32] Xie Y.F., Cai J.Y., Wu Y.R., Hao X.B., Niu S.W., Yin X., Pei Z.B., Wang G.M. (2021). Atomic disorder enables superior catalytic surface of Pt-based catalysts for alkaline hydrogen evolution. ACS Mater. Lett..

[33] Duan B., Liu F., He M., Zhang L.N. (2014). Ag-Fe_3_O_4_ nanocomposites@chitin microspheres constructed by in situ one-pot synthesis for rapid hydrogenation catalysis. Green Chem..

[34] Zheng J., Yang L., Lei H., Wen H.G., Pei K.S. (2019). A facile method to synthesize Pt-Ni octahedral nanoparticles with porous and open-structure features for enhanced oxygen reduction catalysis. ACS Sustain. Chem. Eng..

