# Supplementary material for: Fabrication of Biomass Derived Pt-Ni Bimetallic Catalyst and Its Selective Hydrogenation for 4-Nitrostyrene
Source: Nanomaterials (Basel). 2022 Aug 27;12(17):2968. doi: 10.3390/nano12172968 (PMC9457902; doi:10.3390/nano12172968)
Supplement: Supplementary file 1 [file nanomaterials-12-02968-s001.zip › nanomaterials-1846034-supplementary.pdf]

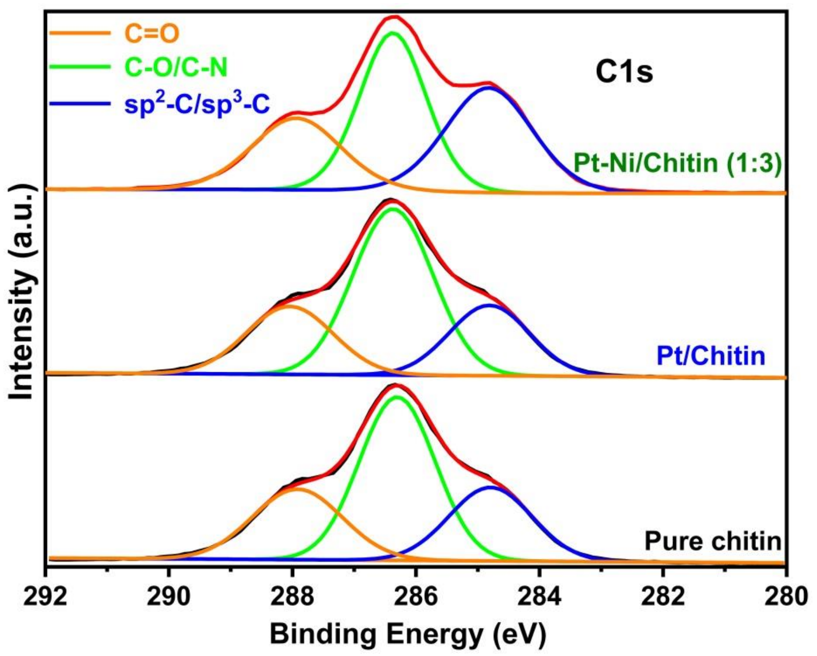

Figure S1. XPS spectra of C 1s for pure chitin, Pt/Chitin and Pt-Ni/Chitin.

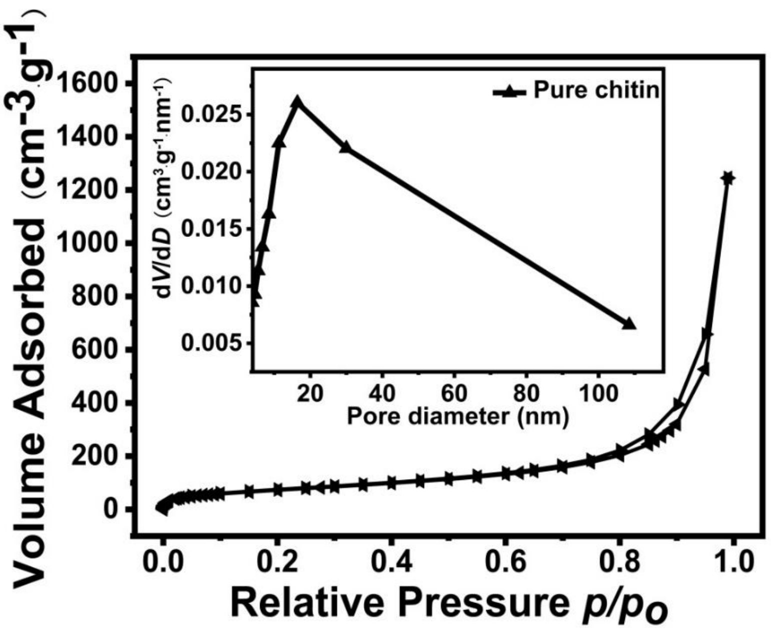

Figure S2. Nitrogen adsorption & desorption isotherms and BJH pore-size distribution of the chitin microspheres.

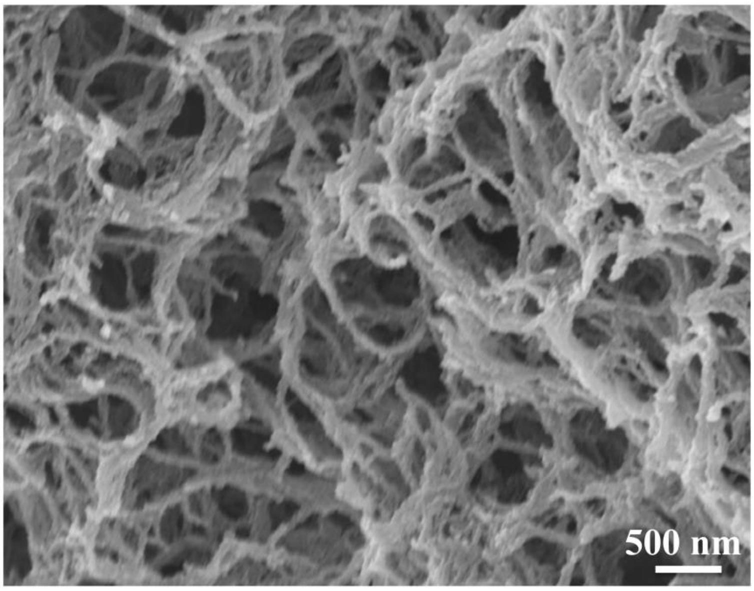

Figure S3. SEM image of the Pt-Ni/Chitin catalyst.

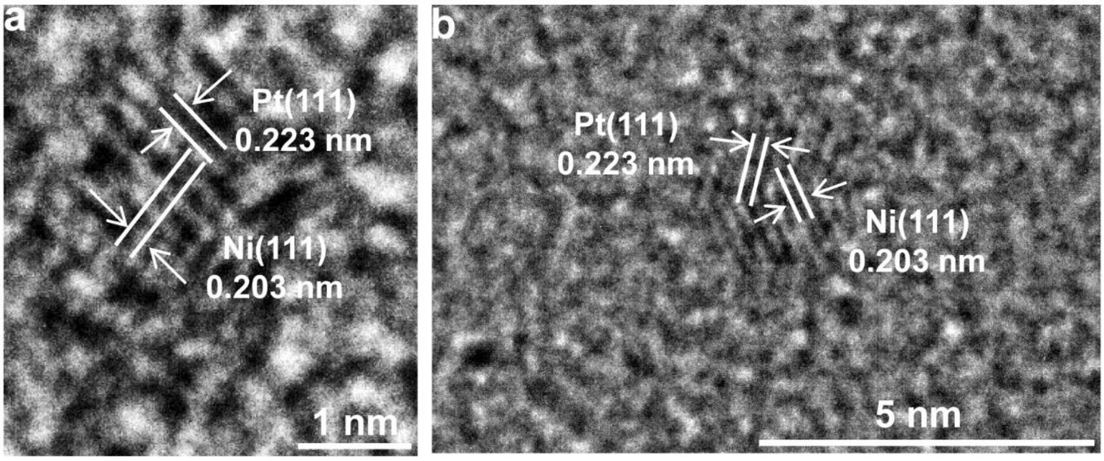

Figure S4. HR-TEM images of an individual Pt-Ni alloy particle (a and b).

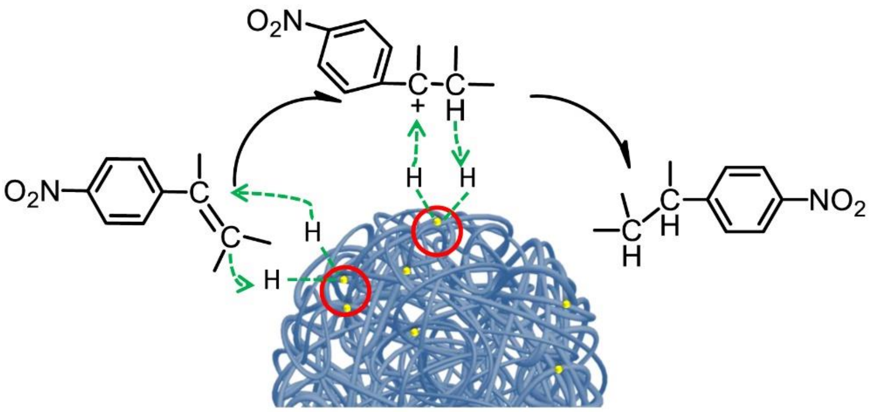

Figure S5. The probable mechanism for the selective hydrogenation of C=C to C–C for 4-nitrostyrene.

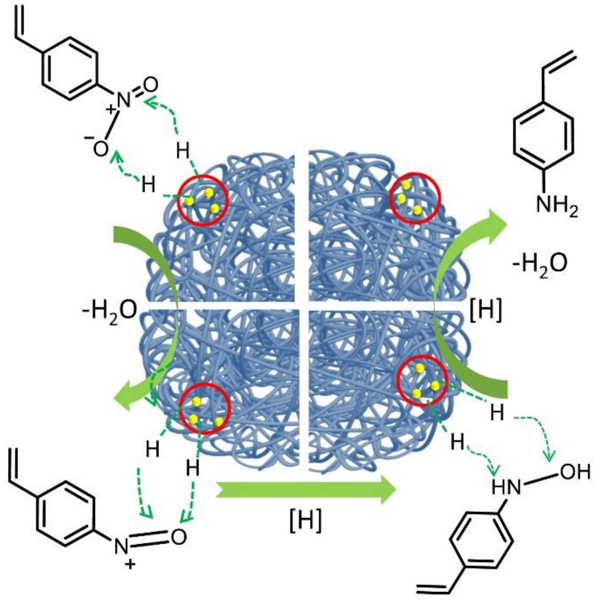

Figure S6. The probable mechanism for the selective hydrogenation of NO_2 to NH_2 for 4-nitrostyrene.

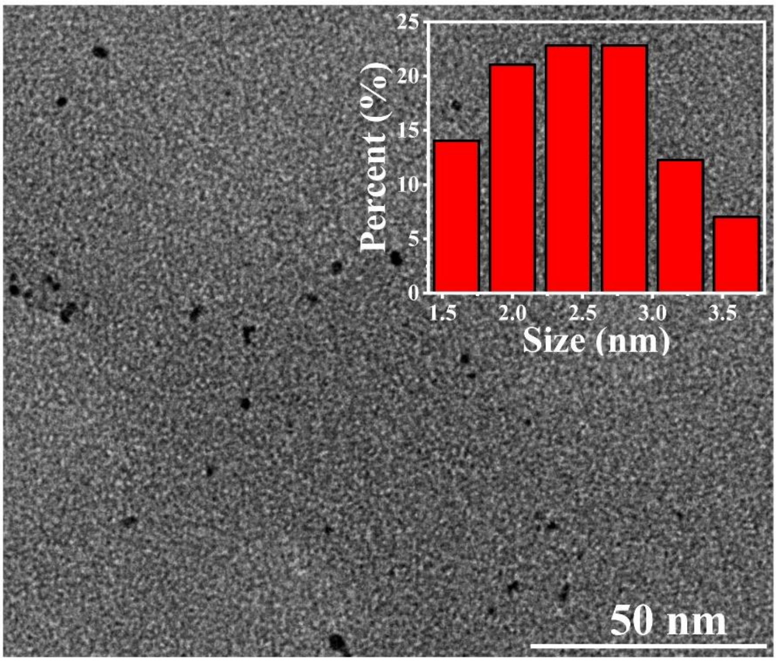

Figure S7. TEM image of the Pt-Ni/Chitin after 5 runs, inset it with the particle size distribution.

Table S1. Hydrogenation of 4-nitroethylene in various reaction conditions.[a]

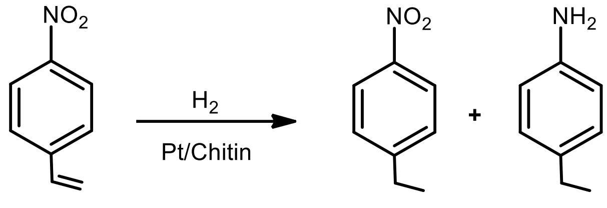

1a 1b 1c

Entry	Solvent	Time (h)	T (°C)	Yeild[b] (%)
1	H_2O	36	25	Trace
2	Toluene	36	25	-
3	Formaldehyde	36	25	-
4	DMF	36	25	Trace
5	THF	36	25	-
6	DCM	36	25	-
7	MeOH	36	25	12
8	IPA	36	25	89
9	IPA	36	45	84
10	IPA	36	60	59
11	IPA	36	90	23

[a]Reaction conditions: 4-nitrostyrene (0.5 mmol), catalyst (4 mg), solvent (5 mL), H_2 (1 bar). [b]b was the yield of 1b, and the yield was GC yield.

Table S2. Some common non-precious metal catalysts for hydrogenation of 4-nitroethylene.[a]

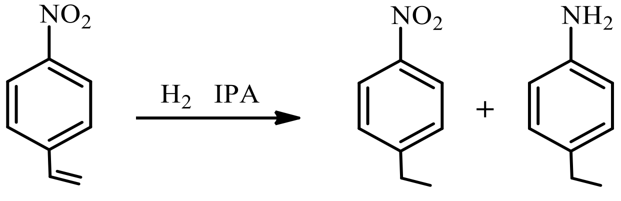

1a 1b 1c

Entry	Catalyst	Time（h）	Yeild[1b]（%）	Yeild[1c]（%）
1	Fe/Chitin	24	-	-
2	Co/Chitin	24	-	-
3	Cu/Chitin	24	-	-
4	Ni/Chitin	24	-	-

[a]Reaction conditions: 4-nitrostyrene (0.5 mmol), catalyst (4 mg), solvent (5 mL), H_2 (1 bar) at room temperature. [b,c] b was the yield of 1b, c was the yield of 1c, The yield was GC yield.

Table S3. Some commercial catalysts for hydrogenation of 4-nitrostyrene.[a]

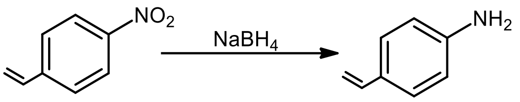

Entry	Catalyst	Temperature (°C)	Time (h)	Yield[b] (%)
1[c]	Commercial nano-Pt	60	3	Trace
2[d]	Commercial Pt/C	60	3	51
3[e]	$H_2PtCl_6·6H_2O$	60	3	57

[a]Reaction conditions: 4-nitrostyrene (0.5 mmol), catalyst (4 mg), solvent (5 mL), $NaBH_4$ (2 mmol), temperature at 60 °C. [b]The yield was GC yield. [c,d,e]The [Pt] content in these catalyst was the same as Pt-Ni/Chitin (3:1).

Table S4. Effect of different ratios of Pt-Ni on the hydrogenation of 4-nitrostyrene.[a]

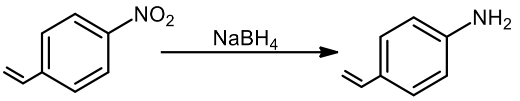

Entry	Catalyst	Temperature (°C)	Time (h)	Yield[b] (%)
1	Pt-Ni/Chitin (3:1)	60	3	99
2	Pt-Ni/Chitin (1:1)	60	3	86
3	Pt-Ni/Chitin (1:3)	60	3	85

[a]Reaction conditions: 4-nitrostyrene (0.5 mmol), catalyst (4 mg), solvent (5 mL), $NaBH_4$ (2 mmol), temperature at 60 °C. [b]The yield was GC yield.

Table S5. The nano-metal content in Pt-Ni/Chitin (3:1) catalyst.

	Initially	1st	2nd	3th	4th	5th
Pt (wt %)	1.21	1.21	1.19	1.18	1.16	1.12
Ni (wt %)	0.39	0.38	0.38	0.37	0.37	0.36
